# Asymmetry in serial femtosecond crystallography data

**DOI:** 10.1107/S2053273316018696

**Published:** 2017-01-30

**Authors:** Amit Sharma, Linda Johansson, Elin Dunevall, Weixiao Y. Wahlgren, Richard Neutze, Gergely Katona

**Affiliations:** a Department of Chemistry and Molecular Biology, University of Gothenburg, Box 462, Gothenburg 40530, Sweden; b Department of Chemistry, Bridge Institute, University of Southern California, Los Angeles, CA 90089, USA

**Keywords:** ex-Gaussian distribution, serial femtosecond crystallography, Bragg reflections, systematic absences, intensity distribution

## Abstract

Serial crystallography is an increasingly important approach to protein crystallography that exploits both X-ray free-electron laser (XFEL) and synchrotron radiation. Serial crystallography recovers complete X-ray diffraction data by processing and merging diffraction images from thousands of randomly oriented non-uniform microcrystals, of which all observations are partial Bragg reflections. Random fluctuations in the XFEL pulse energy spectrum, variations in the size and shape of microcrystals, integrating over millions of weak partial observations and instabilities in the XFEL beam position lead to new types of experimental errors. The quality of Bragg intensity estimates deriving from serial crystallography is therefore contingent upon assumptions made while modeling these data. Here it is observed that serial femtosecond crystallography (SFX) Bragg reflections do not follow a unimodal Gaussian distribution and it is recommended that an idealized assumption of single Gaussian peak profiles be relaxed to incorporate apparent asymmetries when processing SFX data. The phenomenon is illustrated by re-analyzing data collected from microcrystals of the *Blastochloris viridis* photosynthetic reaction center and comparing these intensity observations with conventional synchrotron data. The results show that skewness in the SFX observations captures the essence of the Wilson plot and an empirical treatment is suggested that can help to separate the diffraction Bragg intensity from the background.

## Abstract 2

Distribution analysis of intensity observations in serial femtosecond crystallography data processing helps to separate Bragg reflections from the background detector response.

## 1. Introduction   

X-ray free-electron lasers (XFELs) are linear accelerator based X-ray sources that deliver a peak X-ray brilliance a billion times greater than synchrotron radiation (Emma *et al.*, 2010 ▸). These revolutionary machines were foreseen to create new possibilities in life science (Neutze *et al.*, 2000 ▸) and over the six years since XFELs have been available to users the rapid pace of development has been impressive (Schlichting, 2015 ▸). One major application of XFEL radiation is the development of serial femtosecond crystallography (SFX). A proof-of-principle study first performed at low resolution using crystals of photosystem I (Chapman *et al.*, 2011 ▸) was shortly afterwards extended to high resolution (Boutet *et al.*, 2012 ▸) and has since been applied to time-resolved X-ray diffraction (Tenboer *et al.*, 2014 ▸), the study of protein–protein receptor complexes (Kang *et al.*, 2015 ▸) and *de novo* phasing (Barends *et al.*, 2014 ▸). Serial crystallography has also since been applied to studies using synchrotron radiation (Nogly *et al.*, 2015 ▸) and is expected to become a broadly applied method at storage ring based microfocus beamlines.

Classical synchrotron-based protein crystallography records diffraction images by rotating a protein crystal within an X-ray beam and thereby samples data from all unique reflection planes. In contrast, SFX is a random sampling method whereby complete X-ray data are obtained by processing diffraction images collected from thousands of randomly oriented microcrystals as they are continuously streamed across the path of a highly intense XFEL beam (Chapman *et al.*, 2006 ▸). This strategy creates new challenges and the last few years have seen the emergence of various experimental and data processing tools designed to handle such nuances. *CrystFEL* (White *et al.*, 2012 ▸) is an SFX data analysis software suite which performs indexing by calling *DirAx* (Duisenberg, 1992 ▸), *XDS* (Kabsch, 2010 ▸) and *MOSFLM* (Leslie, 2006 ▸). *CrystFEL* then models diffraction spots as circular regions surrounded by an annulus masking background pixels, similar to early versions of *Denzo* (Otwinowski & Minor, 1997 ▸). Diffraction intensities are quantified by integrating over all pixels occupied within the selected regions, and the spot intensity is calculated either by subtracting the total peak counts from that estimated from the background region, or by using profile fitting (Rietveld, 1969 ▸).

A Monte Carlo merging approach in *CrystFEL* calculates merged intensity for the unique reflections by taking a simple mean of all the symmetrically equivalent observations (Kirian *et al.*, 2010 ▸). This is appropriate when the corresponding intensities follow a unimodal normal distribution. An alternative to *CrystFEL* which is based upon an extrapolation method assumes that all the crystals in an SFX experiment are identical and the final energy distribution in the reciprocal space is Gaussian (Zhang *et al.*, 2014 ▸). Microcrystals possess very different diffraction power and the detector distance cannot be optimized for each crystal as in a conventional synchrotron-based experiment. The data collection parameters are instead optimized to capture the highest-resolution reflections from the best ordered crystals. This also means that many high-resolution reflections without signal are integrated from the large body of weakly diffracting crystals. Although attempts are made to limit the integration radius to that of the crystal resolution, this point estimate is error prone. As a result, intensity observations may not necessarily follow a single normal distribution.

Here, we explore an alternative to the perfect Gaussian distribution for serial crystallography data using data recorded at the LCLS (Linac Coherent Light Source, Stanford University, California) from microcrystals of the *Blastochloris viridis* (*B. viridis*) photosynthetic reaction center (RC_vir_) as a representative example. We examine the asymmetry in the reflection intensities using an ex-Gaussian distribution as a minimal extension of a Gaussian distribution and contrast the asymmetry in SFX data to more conventional X-ray diffraction observations from a single crystal collected at a synchrotron source. Finally, we make a preliminary proposal for how the properties of an ex-Gaussian distribution may be exploited to infer the Bragg intensities of an ‘idealized crystal’ in the microcrystal slurry.

## 2. Materials and methods   

### 2.1. Growth and purification of reaction center from *B. viridis*   

The photosynthetic reaction center from *B. viridis* was cultivated and purified as described by Johansson *et al.* (2013 ▸).

### 2.2. Lipidic sponge phase batch crystallization   

Lipidic sponge phase was prepared as described by Wöhri *et al.* (2009 ▸) with the following modifications. Melted monoolein was thoroughly mixed in a ratio of 3:2 (*v*/*v*) with 0.1 *M* HEPES pH 7.5, 0.1% LDAO until a viscous transparent lipidic cubic phase was obtained. The formed phase was then transferred into a glass vial and sponge-phase-inducing solution (1:4 ratio) was added containing 16% Jeffamine M-600, 1 *M* HEPES pH 7.9, 0.7 *M* ammonium sulfate, 2.5% 1,2,3-heptanetriol, which swells the cubic phase to the sponge phase. After phase separation overnight, the upper phase (sponge phase) was harvested. Crystals were grown using batch crystallization in the lipidic sponge phase. Equal amounts of sponge phase and protein were mixed in a 4:1 ratio (*v*/*v*) with 1.2 *M* tris-sodium citrate (2 parts sponge phase, 2 parts protein, 1 part tris-sodium citrate) and allowed to incubate for several weeks. Crystals grew in the dark at room temperature and were delivered to the CXI (coherent X-ray imaging) instrument using a sample loop. The protein concentration used for crystallization setups was 20 mg ml^−1^ with an optical purity ratio of *A*_280_/*A*_830_ = 2.27.

### 2.3. Synchrotron single-crystal data collection   

Single-crystal X-ray data were collected under cryo-conditions with a 225 mm MarMOSAIC CCD detector at ID23-2 at the ESRF (Grenoble, France) (λ = 0.8726 Å). The oscillation range per image was 1°, the exposure time 1.0 s, the distance to the detector 373.1 mm. The crystals belong to the space group *P*2_1_2_1_2_1_ with unit-cell dimensions of *a* = 57.6, *b* = 82.9, *c* = 382.1 Å, α = β = γ = 90°.

### 2.4. SFX data processing and analysis   

RC_vir_XFEL_ data from 1175 images were indexed and integrated using the *indexamajig* program of the *CrystFEL* 0.6.2 software suite. Data were indexed using the space group *P*2_1_2_1_2_1_ with unit-cell dimensions of *a* = 57.9, *b* = 84.8, *c* = 384.3 Å, α = β = γ = 90° and data reflections were integrated up to the apparent diffraction limit of each crystal by using the _rescut _push-res = 0 option. Three parallel data processing approaches, namely *CrystFEL process_hkl*, *CrystFEL partial­ator* and the Ideal Crystal approach, were used to merge data. The *CrystFEL process_hkl* scaled and merged the data using the Monte Carlo method; the *CrystFEL partialator* method was used to merge the data using the partiality ‘scgaussian’ model with data scaling using three iterations. Thirdly, the Ideal Crystal approach which is based on the Markov chain Monte Carlo (MCMC) method (Gilks *et al.*, 1996 ▸) was implemented using the *pymc3* library (Salvatier *et al.*, 2016 ▸) and the unmerged data which were sorted and arranged in the group of unique reflections using the *cctbx* libraries (Sauter *et al.*, 2013 ▸). The data collection and processing statistics using all three approaches are summarized in Table 1 ▸. Wilson *B* factors for the Ideal Crystal approach were matched with those of *CrystFEL process_hkl *and *Cryst­FEL partialator* approaches by multiplying the intensities and σ’s by a correction factor [*f* = *b* − (*b* − 1)*q* ^2^/*q* ^2^_*n*_, where *b* is a variable, *q* is the inverse resolution squared (1/*d* ^2^) and *q_n_* is the *q* of the highest-resolution reflection]. Two sets of the Ideal Crystal data were generated using correction factors with *b* = 1.05 and *b* = 1.19 in order to compare with the *CrystFEL process_hkl* and *partial­ator* approach, respectively. All four data sets were processed using *CCP4* 7.0.020 (Winn *et al.*, 2011 ▸) *truncate* software (French & Wilson, 1978 ▸).

For structural comparison between the Ideal Crystal data sets and those from *CrystFEL* data processing approaches, all four diffraction data sets were cut at 3.5 Å resolution. Mol­ecular replacement (MR) solutions were searched in the *P*2_1_2_1_2_1_ space group with a search model of RC_vir_ (PDB entry 4cas, Johansson *et al.*, 2013 ▸) using *Phaser-MR* in *Phenix* version 1.10-2155-1692 (Adams *et al.*, 2010 ▸). The MR solutions using Ideal Crystal data sets showed the higher log-likelihood gains among the tested data processing strategies (shown in Table 1 ▸). RC_vir_XFEL_ structures were refined for all four data sets using the same refinement strategies in the *Phenix* suite. The refinement strategy implemented was performed using five cycles of refinement for coordinates, real space, rigid body and individual *B* factors. The refinement protocol was further modified by using maximum likelihood target functions and the best weights for the X-ray target functions, and the *B*-factor restraints were optimized. Water update and automatic correction of N/Q/H errors were kept active during the refinement. Refinement statistics are summarized in Table 1 ▸ which shows that refinement parameters are quite similar between the corresponding Ideal Crystal and *CrystFEL* approaches. Simulated annealing composite omit maps were calculated in *Phenix* for all four data sets. All the maps were contoured at 1σ.

## 3. Results   

### 3.1. Overview of data processing steps   

The goal of any crystallographic analysis of diffraction patterns is to present the data in a way that the structural refinement packages can use. The new concept of serial crystallography (Chapman *et al.*, 2011 ▸) deals with very high multiplicity, but the underlying principles are the same as in traditional approaches to crystallography in that serial crystallography records multiple observations for any unique reflection from multiple images, and then merges these observations to yield *I*_*hkl*_ and σ_*hkl*_ which scaling and other crystallographic programs can use. The data processing scheme used here starts with the indexing and integration of the diffraction images. Complete diffraction data are then obtained in the form of a stream file containing the list of all the observations. These observations are then mapped to the asymmetric unit using the *cctbx* Python libraries (Grosse-Kunstleve *et al.*, 2002 ▸). The key scientific idea here is to determine the distribution of the observations, and the histogram for each reflection is calculated and fitted with different distribution functions. The fitting parameters are then used to diagnose and determine the Bragg reflection and diffuse intensity response.

### 3.2. Symmetric and asymmetric distribution functions   

Intensity distributions for all the unique reflections were calculated and fitted using selected distribution functions. Standard approaches of processing crystallographic data assume an idealized Gaussian distribution function:where μ and σ provide information about the mean and variability in the reflection, respectively. Serial crystallography risks merging many more weak observations than expected when using classical data collection strategies and therefore the distribution of observations may be expected to be skewed towards a larger number of weak observations. To test this possibility, we also consider a minimal extension of the Gaussian distribution known as an ex-Gaussian distribution function which is a convoluted distribution of a Gaussian and an exponential distribution function and is represented as:where erf is an error function with erf(0) = 0; erf(∞) = 1. Here, μ and σ provide information on localization and variability of the Gaussian part, respectively, and τ is the mean of an exponential component, providing information about the degree of skewness in the distribution of reflection intensities.

A parameter search algorithm using maximum likelihood estimation (Lacouture & Cousineau, 2008 ▸) was used to recover optimal parameters for both Gaussian and ex-Gaussian distributions to crystallographic observations for any given *hkl* Miller indices. For the parameter value (*p*) which allows a correct fit of the distribution function *f*(*x*|*p*) to the data (*x*), the minus log-likelihood criterion was applied in which the minimum of a −LogL is estimated, where LogL for a parameter value *p* is defined as


Scanning through all possible values of the parameter is time consuming, specifically when fitting with multi-parameter distribution functions. Therefore, as a first approximation the local minimum of the function was reached by iteratively adjusting the starting parameter values using the Simplex method based on the steepest gradient algorithm (Lacouture & Cousineau, 2008 ▸). We later extend the inference within the *a posteriori* parameter space using the MCMC algorithm (Gilks *et al.*, 1996 ▸) to estimate the uncertainty in distribution parameters.

### 3.3. Expectation value calculation using XFEL data   

X-ray diffraction images were collected by injecting thousands of microcrystals of RC_vir_ into the XFEL pulses coming with a repetition rate of 120 Hz at the LCLS. Images were indexed and integrated using the *indexamajig* program of the *CrystFEL* suite (White *et al.*, 2012 ▸). All the indexed observations for each microcrystal are collectively put together into a complete diffraction data set in one stream file. Each batch contains a set of partial reflections which were re-assembled to have all the equivalent reflections in an order. All the equivalent observations were then mapped to the asymmetric unit of space group *P*2_1_2_1_2_1_ using the *cctbx* Python library through the map_to_asu function of Miller array objects.

**Figure 1:**
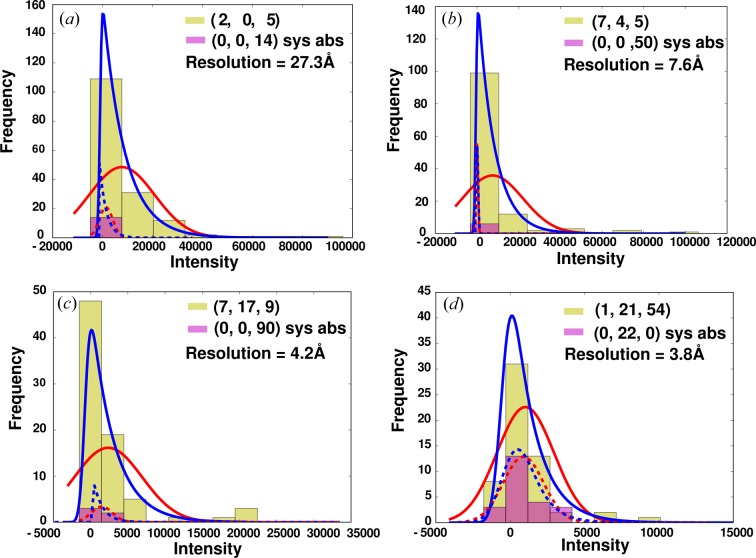
(*a*)–(*d*) Histograms for the distribution of some of the unique Bragg reflections (yellow) and the systematically absent reflections (magenta) selected from the resolution shells around 27.3, 7.6, 4.2 and 3.8 Å, respectively, from the RC_vir_XFEL_ data. The fits to these distributions using a Gaussian (red) and an ex-Gaussian (blue) function are shown. The fits to the Bragg reflections and the systematically absent reflections are shown as full and dashed lines, respectively. Miller indices for the reflections used are given in parentheses.

XFEL diffraction RC_vir_ data (RC_vir_XFEL_) were considered up to 3.5 Å resolution as previously described (Johansson *et al.*, 2013 ▸). We calculated the histogram for the distribution of observations for each reflection and fitted it with both a Gaussian and an ex-Gaussian distribution profile. Figs. 1 ▸(*a*)–1 ▸(*d*) show the Gaussian (red) and the ex-Gaussian (blue) fits to the histograms for four reflections selected at different resolutions. The ex-Gaussian fit was observed to provide a better shape fit to the histogram than the Gaussian fit. Table 2 ▸ provides the values of the fitting parameters obtained using the two distributions for the selected reflections. μ_g_ and σ_g_ are the fitting parameters obtained using the Gaussian fit whereas μ_exg_, σ_exg_ and τ_exg_ are the ones obtained using the ex-Gaussian fit. Consistent with the definition of an ex-Gaussian distribution, we observed that for the reflections which have relatively reduced skewness the ex-Gaussian approaches a normal distribution, as is seen for the reflection (1, 21, 54) in Fig. 1 ▸(*d*). In contrast, for the reflection (7, 4, 5) in Fig. 1 ▸(*b*), where skewness is high, the ex-Gaussian distribution approaches an exponential distribution.

**Figure 2:**
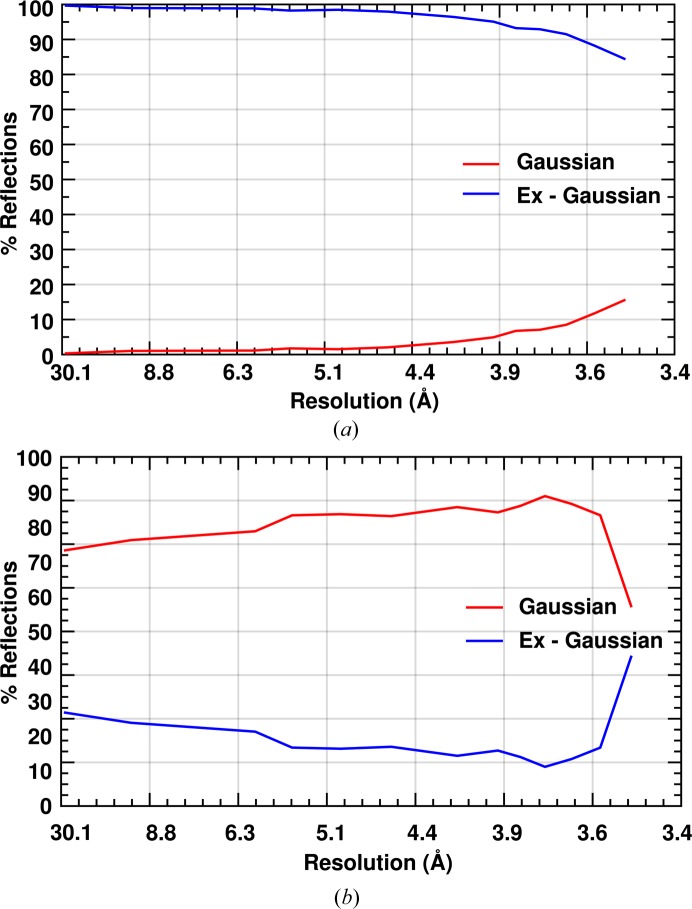
Percentage of reflections from (*a*) RC_vir_XFEL_ and (*b*) RC_vir_Sync_ data that could be best explained using a Gaussian (red) and an ex-Gaussian (blue) distribution.

A Gaussian is a subset of an ex-Gaussian function; hence a fit with an ex-Gaussian will always be better than or equal to that of a Gaussian. In order to check the desirability of such a distribution function to correctly model the frequency of intensity observations, we performed a likelihood ratio test for nested functions. In this test, twice the difference between the negative of the log-likelihood of the Gaussian and ex-Gaussian provides the chi-square estimate for an extra degree of freedom. Chi-square values for each reflection were calculated and the decision to fit the reflection using an ex-Gaussian distribution profile was taken on the basis of 5% chance criteria, which gives a chi-square cutoff score of 3.84 for a single degree of freedom. For RC_vir_XFEL_ data, it was found that 95% of reflections strongly favour an estimation to be made using an ex-Gaussian distribution function. This preference for an ex-Gaussian over a Gaussian fit was found to be consistent over the complete resolution range (Fig. 2 ▸ *a*).

**Figure 3:**
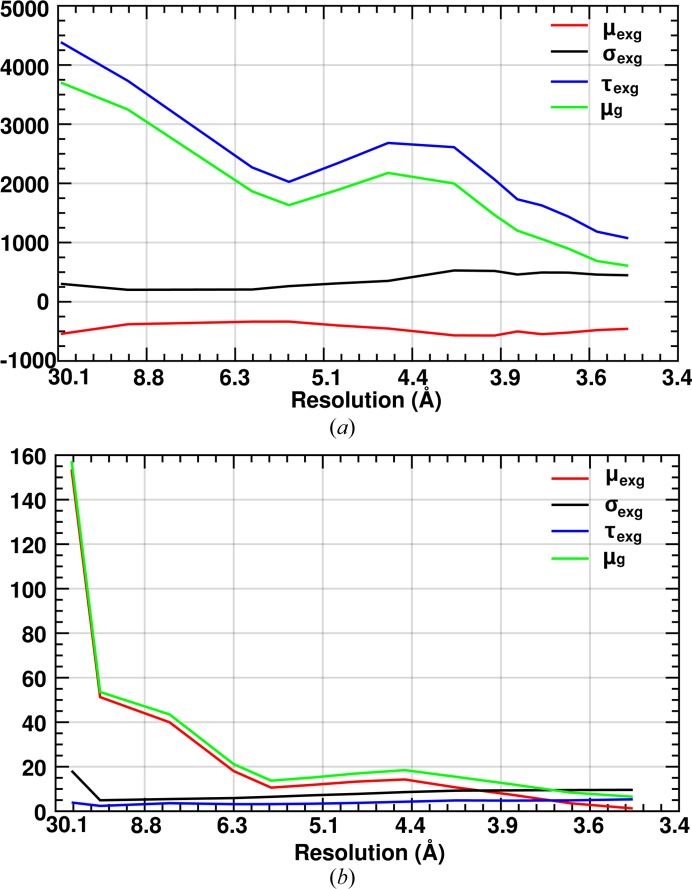
This shows the variation in the mean (μ_exg_, red) of the Gaussian part of an ex-Gaussian fit, the standard deviation (σ_exg_, black), the skewness mean (τ_exg,_ blue) from the exponential part of the ex-Gaussian fit and the mean intensity of the reflection (μ_g_, green) with the resolution for (*a*) RC_vir_XFEL_ and (*b*) RC_vir_Sync_ data.

To explore further the information contained in a Gaussian and the exponential parts of an ex-Gaussian distribution, we plotted the mean (μ_exg_) and the skewness (τ_exg_) parameters of the ex-Gaussian function as a function of resolution (Fig. 3 ▸ *a*). At high resolution (above 7 Å), τ_exg_ follows the Wilson distribution in a similar manner to μ_g_ of the Gaussian function. τ_exg_ and μ_g_ have the same magnitude in the high-resolution range and show standard maxima and minima at 4.5 Å and 6 Å, respectively. On the other hand, μ_exg_ does not appear to follow the expected Wilson intensity distribution at high resolution.

### 3.4. Comparison with the systematic absences   

The magenta and yellow coloured histograms in Figs. 1 ▸(*a*)–1 ▸(*d*) show the distributions of the four systematically absent and non-absent Bragg reflections, respectively, in different resolution ranges, 27.3, 7.6, 4.2 and 3.8 Å. Comparing the histograms of the Bragg reflections at high resolutions with those of the systematically absent reflections in our data, we observe that the most frequent intensity observations of non-absent Bragg reflections overlap with those of the systematically absent reflections present in the same resolution range. In addition, there is a small fraction of valuable observations that are responsible for the extended tail of the distribution. Curiously, not even at the lowest-resolution reflection (27.3 Å in Fig. 1 ▸ *a*) is the distribution symmetric and we observe that there is no genuine shift in the most frequent observations compared with the systematic absence at similar resolution.

### 3.5. Comparison with synchrotron data   

A similar analysis of reflection intensities was carried out using diffraction data collected at a synchrotron source (ESRF) from a single macrocrystal of RC_vir_. Lipidic sponge phase microcrystals had a different space group from the earlier form of the macrocrystals (Johansson *et al.*, 2012 ▸). To make a fair comparison, we have reproduced an RC_vir_ macrocrystal to match the crystal packing with that of the microcrystals used for XFEL-based data collection. Although we were able to produce macrocrystals in the *P*2_1_2_1_2_1_ space group, they diffracted only up to 3.6 Å in contrast to the 1.86 Å limit achieved earlier by using the RC_vir_ crystals produced in the *P*2_1_2_1_2 space group (Wöhri *et al.*, 2009 ▸). Table 1 ▸ provides the crystallographic data statistics showing the comparison between the data collected at the synchrotron source to 3.6 Å and those collected at the XFEL source at 3.5 Å. The synchrotron data (RC_vir_Sync_) have smaller multiplicity in comparison with the XFEL data.

**Figure 4:**
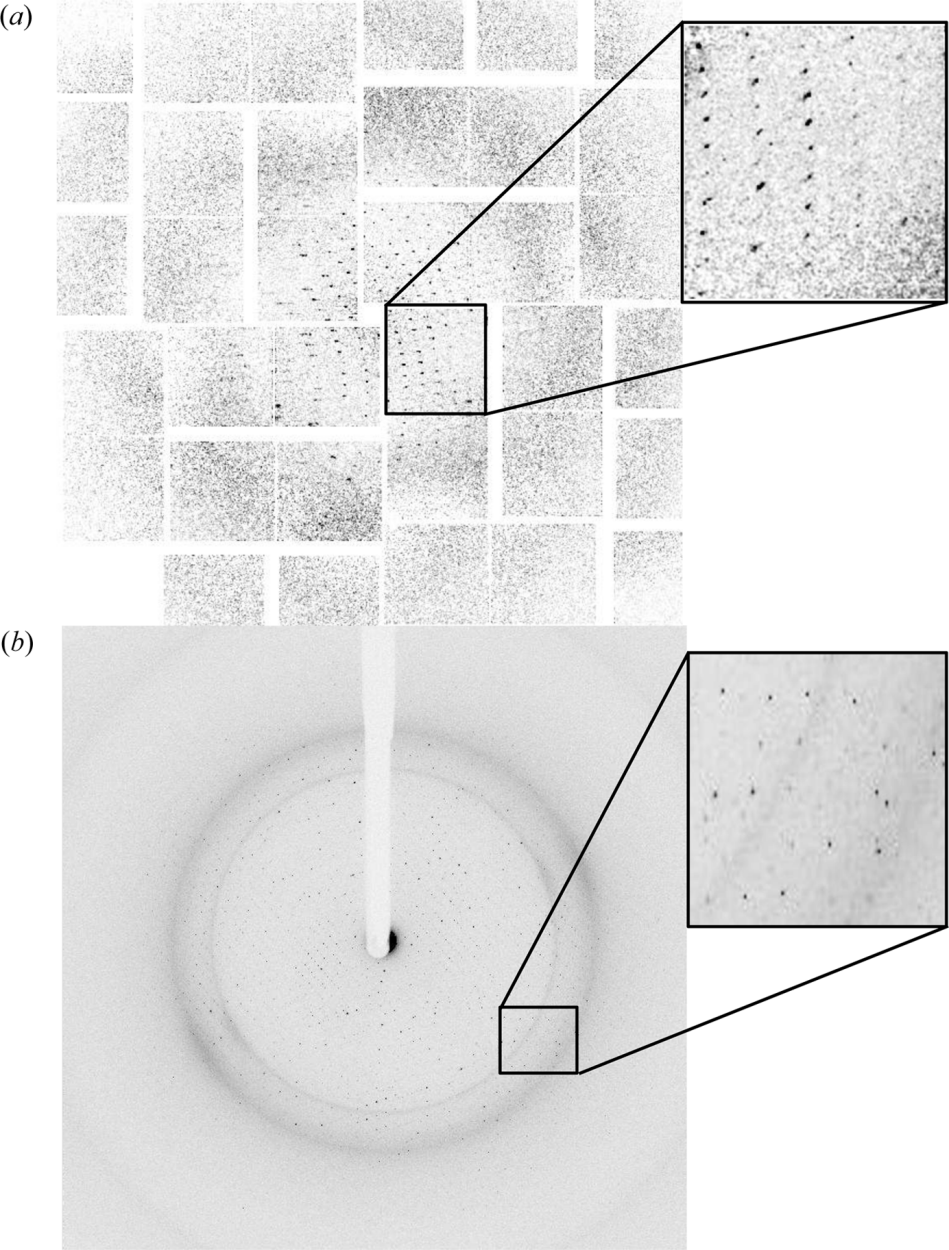
Diffraction quality of RC_vir_XFEL_ and RC_vir_Sync_. A representative diffraction image collected from (*a*) microcrystals of RC_vir_ at the CXI (coherent X-ray imaging) beamline of the LCLS and (*b*) a crystal of RC_vir_ on the ID23-2 beamline of the ESRF.

It is often sufficient that crystals are indexed with the help of their low-resolution reflections, but the measured intensities at predicted spot positions can be practically zero at higher resolution in the case of low-quality crystals. Fig. 4 ▸(*a*) shows an SFX diffraction snapshot from a weakly diffracting crystal where high-resolution reflections are not observed; nevertheless Bragg positions are integrated beyond the visible limit of Bragg spots. In synchrotron crystallographic data collection (Fig. 4 ▸ *b*), a similar large variation in diffraction intensity is not observed unless the diffraction is very anisotropic or the crystal is poorly centered. For this reason, we see that the histogram of synchrotron reflections is more symmetric and can be modeled unambiguously with a Gaussian distribution (Fig. 2 ▸ *b*). If fitted using an ex-Gaussian distribution, the tail is less pronounced and τ_exg_ has little influence on the mean intensity of the reflection (Fig. 3 ▸ *b*). Conversely, in SFX data only a few good-quality crystals produce overall high-intensity observations leading to a tail in the intensity histogram.

### 3.6. Empirical inference of Bragg intensity from idealized crystals   

When developing a statistical method, it is important to define the goal of the analysis. The presumed goal of current practice is to describe the central tendency of the data and the variability of observations. It is expected that partiality correction and image-by-image scaling reduce the variability of observations, but this does not change the original goal. If the distribution is symmetric and unimodal the statistical mean could estimate the most frequent intensity observation and our data show that this is a valid assumption for most of the Bragg intensities recorded from a single crystal of reaction center at a synchrotron. There are two serious problems with the original goals when it comes to SFX data. Firstly, we have shown that the distribution is very asymmetric, the mean is ill suited to describe the central tendency of the distribution and the standard errors determined from such distributions cannot accurately describe the confidence interval of the mean. Secondly, the comparison of systematic absences and non-absent Bragg reflections at similar resolution shows that their mode is essentially the same; therefore, it is potentially misleading to try to determine (more accurately) the most frequent intensity of non-absent Bragg reflections. On the other hand, we are free to choose more meaningful goals for the statistical analysis, for example by replacing the question ‘what is the typical observed Bragg reflection intensity in an experiment?’ with ‘how high can the Bragg intensity be in an experiment?’. While the typical intensity observation is strongly influenced by diffuse scattering, the highest ones are more likely to originate from good crystals. Thus, we can aim to determine the diffraction intensity of an ‘idealized’ crystal in its ideal diffraction condition. The highest-intensity observation of any given unique reflection may or may not originate from this group of ideal crystals at the top of their rocking curve, but we can define a region of the intensity probability distribution where we expect the ideal crystal reflections to appear even in the absence of an actual observation. This depends on how restrictively the ideal region is defined and the multiplicity of observations.

**Figure 5:**
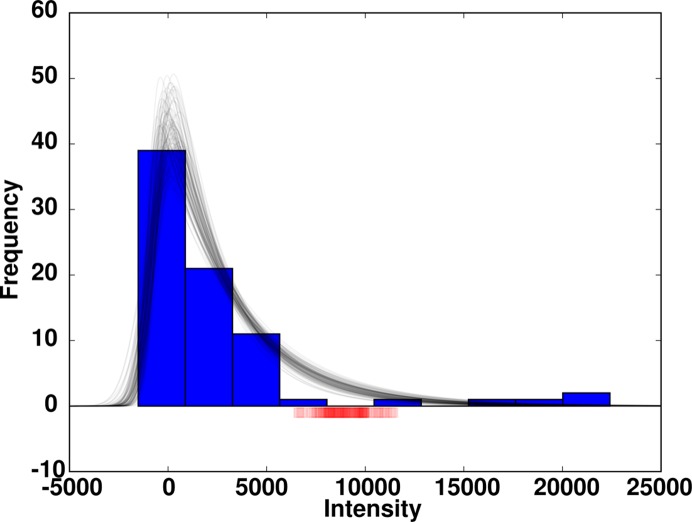
Ex-Gaussian distribution profiles using the Markov chain Monte Carlo *a posteriori* estimates (plots in black) for a Bragg reflection whose intensity histogram is shown in blue. Shown in red is the spread of the intensity value (*I*_ideal_) at which the c.d.f. of ex-Gaussian distributions reaches 0.95.

As a first step, we have developed a Bayesian model to infer the probability distribution of the parameters of the empirical ex-Gaussian distribution from the intensity observations of each Bragg reflection using a minimally informative prior knowledge (assuming a uniform distribution). We have approximated the posterior distribution (given the intensity observations) using the MCMC method (Gilks *et al.*, 1996 ▸) based on a similar probabilistic model that was previously used for determining the structure amplitude difference of correlated intensity observations (Katona *et al.*, 2016 ▸). For each Bragg reflection we determined the maximum *a posteriori* values of model parameters (μ_exg_, σ_exg_, τ_exg_) as a starting point and calculated 50 000 Monte Carlo samples [Metropolis stepping (Metropolis *et al.*, 1953 ▸)], the first 40 000 of which we discarded. We performed thinning of the Markov chain by taking every tenth sample from the posterior joint probability distribution and determined the intensity value (*I*_ideal_) at which the cumulative distribution function (c.d.f.) of each ex-Gaussian distribution sample reached a critical value (0.95). The optimal level of critical cumulative probability can be set empirically, but ideally it should not be less than the expected frequency of the diffuse scattering intensity response. A representative example illustrating the posterior distribution samples and corresponding *I*_ideal_ positions is shown in Fig. 5 ▸. Finally, we have defined the intensity of the idealized crystals as the mean of *I*_ideal_ and its uncertainty as the standard deviation of *I*_ideal_.

**Figure 6:**
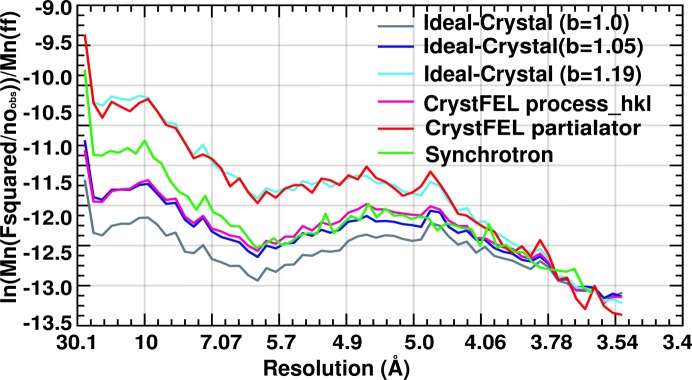
Wilson plots for the RC_vir_XFEL_ data generated using Ideal Crystal (*b* = 1), Ideal Crystal (*b* = 1.05), Ideal Crystal (*b* = 1.19), *CrystFEL* _push-res = 0 *process_hkl* and *CrystFEL partialator* _model = scgaussian options are shown in grey, blue, cyan, magenta and red, respectively. Wilson plot for the RC_vir_Sync_ data is shown in green.

These idealized data were further processed with *truncate* of the *CCP4* package in parallel with the *CrystFEL* *process_hkl* and *partialator* data as reference data sets. The Ideal Crystal approach resulted in a flatter Wilson plot than the data processed by *CrystFEL process_hkl* (15 Å^2^ *versus* 27 Å^2^, respectively). These Wilson plots are comparable with single-crystal synchrotron data recorded to similar resolution (37.6 Å^2^) (Fig. 6 ▸), but the partiality-corrected data set is substantially steeper even if it originates from the same set of observations. This is not very surprising since many traditional crystallographic properties have weaker theoretical foundations in SFX. In particular, the observed Wilson plot cannot be directly related to the random displacement of atoms in the unit cells of a single crystal since each crystal has different diffractive power, mosaic spread, atomic displacement parameter distribution *etc*. Thus, the Wilson plot and the derived Wilson *B* factor lose their traditional physical meaning without further information about the distribution of the microcrystals. This is not necessarily a problem since empirical tools can be employed to approximate the SFX observations to those of single-crystal diffraction or to improve the quality of the electron-density maps or refinement. *Partialator* scaling tries to incorporate a crystal-specific scaling factor and *B* factor to compensate for this heterogeneity in SFX data sets, but the question then becomes ‘what is the optimal ultimate scale target?’.

**Figure 7:**
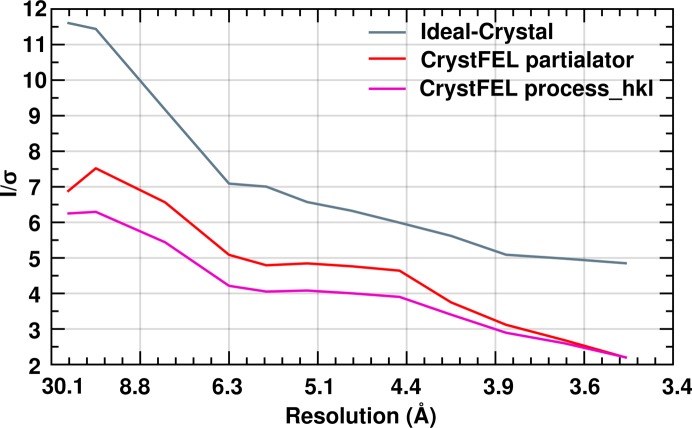
Plots showing *I*/σ *versus* resolution (Å) for the RC_vir_XFEL_ data indexed using _push-res = 0 option of *CrystFEL*. Plots in grey, red and magenta correspond to three different data processing methods – Ideal Crystal, *CrystFEL partialator* _model = scgaussian and *CrystFEL process_hkl*, respectively.

We have applied a correction factor [*b* − (*b* − 1)*q* ^2^/*q* ^2^_*n*_, where *b* is a variable, *q* is the inverse resolution squared (1/*d* ^2^) and *q*_*n*_ is the *q* of the highest-resolution reflection] to the intensities and σ’s prior to the *truncate* step to match the Wilson *B* factor of the Ideal Crystal approach with that of the *process_hkl* and *partialator* data (26.6 and 59.6 Å^2^, respectively, after the correction) to allow a fair comparison. The resulting Wilson plots are shown in Fig. 6 ▸. The *I*/σ (Fig. 7 ▸) determined by the Ideal Crystal approach appears to be higher and reflects an appropriate accuracy of the measurement. This is strongly influenced by the choice of the c.d.f. critical value (0.95).

**Figure 8:**
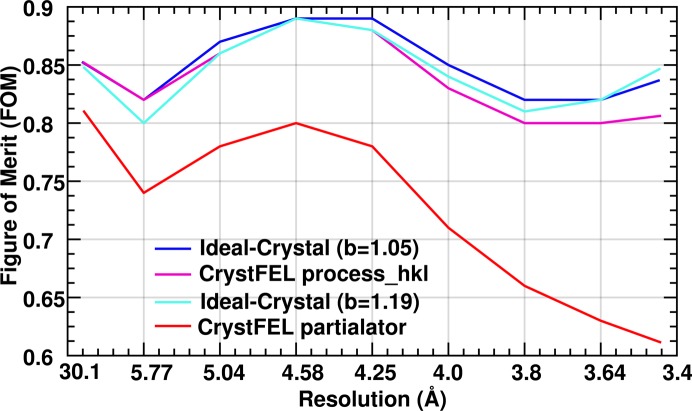
Figure of merit (a measure of phase quality) *versus* resolution plots for the RC_vir_XFEL_ data generated using Ideal Crystal (*b* = 1.05), *CrystFEL process_hkl*, Ideal Crystal (*b* = 1.19) and *CrystFEL partialator* _model = scgaussian options are shown in blue, magenta, cyan and red, respectively.

Subsequent modeling steps and map calculations were performed in the *Phenix* suite (Adams *et al.*, 2010 ▸) using identical protocols (see §2). After molecular replacement by *Phaser* the final log-likelihood gain was higher when using the Ideal Crystal data (Table 1 ▸). The best *R*_free_ value was obtained from the Ideal Crystal approach when scaled to the *partialator* data, but at the same time the omit-map quality became worse, although not to the same extent as seen in the *partialator* data. When scaled to the *process_hkl* data the *R*_free_ was slightly lower using the Ideal Crystal approach than *process_hkl* (27.3% *versus* 27.4%), and the resulting figures of merit, which provide a measure of phase quality for each reflection, were higher (Fig. 8 ▸).

**Figure 9:**
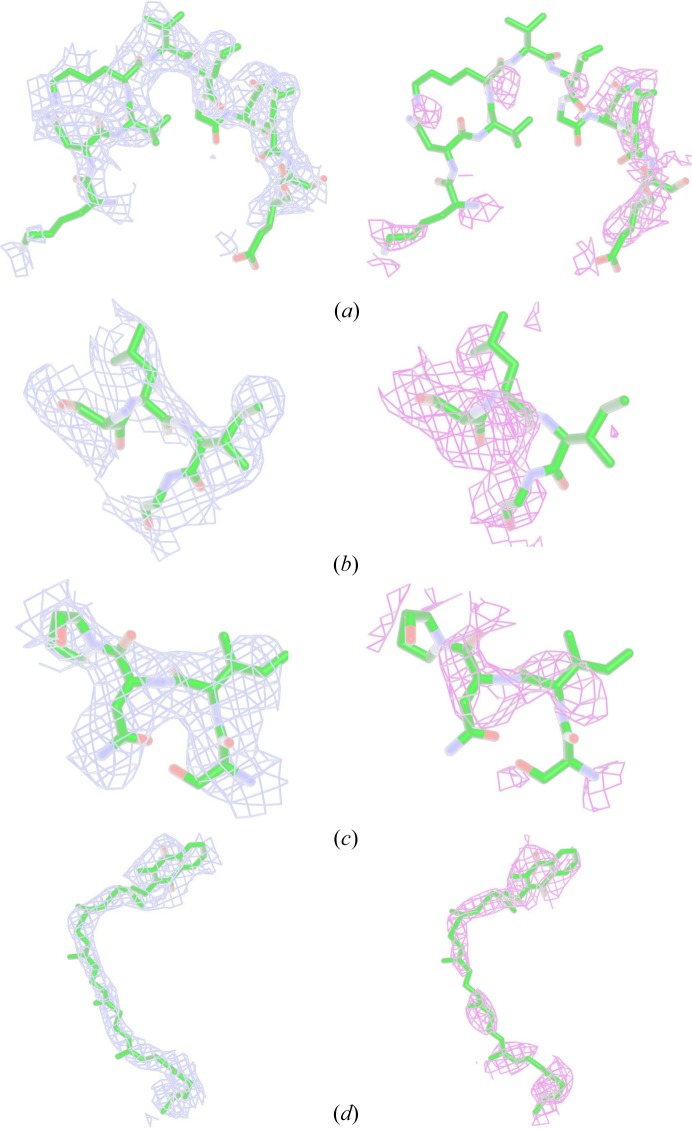
Simulated annealing composite omit maps calculated using RC_vir_XFEL_ data sets processed using the Ideal Crystal (*b* = 1.05) approach (blue) and *CrystFEL process_hkl* option (magenta). Selected regions of RC_vir_ subunits are shown in four different panels: (*a*) residues 57–67 in cytochrome C subunit, (*b*) residues 47–50 in the intra-membrane subunit L, (*c*) residues 65–68 in the intra-membrane subunit L and (*d*) the mena­quinone in subunit M. Residues are shown in element colour.

Fig. 9 ▸ presents a comparison of simulated annealing omit maps calculated from the two data sets – Ideal Crystal (*b* = 1.05) and *process_hkl*. Map comparison [Ideal Crystal (blue) *versus* *process_hkl* (magenta)] for four selected regions of RC_vir_, *i.e.* residues 57–67 in cytochrome C subunit, residues 47–50 in the intra-membrane subunit L, residues 65–68 in the intra-membrane subunit L and the mena­quinone in subunit M, is shown. In summary, the *process_hkl* and Ideal Crystal data sets yield electron-density maps and other quality indicators that are broadly comparable, with the Ideal Crystal data sets in some respects seeming better, without any extensive optimization.

It will ultimately be necessary to distinguish distributions that are likely to contain at least one Bragg intensity observation from those that do not contain any. Although one may expect that the best Bragg reflections are at the high end of the c.d.f., the converse is not necessarily true and some observation distributions are presumed to not contain any measurable Bragg reflections at all. In the absence of a genuine observation a prior estimate has to be used. These efforts may be greatly facilitated by the intensity observations of systematic absences as they can help to define the low end of the dynamic range at each resolution bin. As a further development, it may also be possible to optimize the choice of the critical c.d.f. probability: at a higher value one may expect even better selectivity, at the expense of increased uncertainty of *I*_ideal_. One may also find better ways to analyze the posterior distribution of *I*_ideal_ (reporting the median or mode) and alternative ways to describe their dispersion (for example, using median absolute deviation). Our procedure does not necessitate the use of an ex-Gaussian distribution; in principle, any asymmetric distribution (uni- or multimodal) may be applied. Once the most successful empirical distribution is identified, a suitable outlier recognition method can be developed. Since genuine Bragg intensity observations may be a small fraction of the multiplicity, it may be beneficial to incorporate more *a priori* information in the Bayesian model (using empirical Bayes methods or from first principles).

## 4. Conclusion   

SFX experiments can produce highly heterogeneous data and our primary purpose was raising the awareness of their incorrect treatment. We observed that in the SFX data the skewness of the intensity observations follows the characteristic trend of a protein Wilson plot; the same is not true for the intensity of the most frequent observations. The most frequent intensity observations do not appear to originate from Bragg reflections of protein. To deal with the weak diffraction images one possibility is to pre-filter the reflection observations using a dynamic resolution cutoff per image basis. This approach risks rejecting weak observations that otherwise contain useful information, and indeed we have not observed a sharp distinction between the diffuse scattering signal and Bragg reflections. Instead, the information content can be judged from the distribution of the entire data set and the described ex-Gaussian diagnostics and our intensity inference strategy is a step in this direction.

## Supplementary Material

PDB reference: 5m7k

PDB reference: 5m7j

PDB reference: 5m7l

## Figures and Tables

**Table 1:** Data collection, processing and refinement statistics *M* = Multiplicity for the reflection (*h*, *k*, *l*). LLG = final log-likelihood gain after molecular replacement (MR) by *Phaser*.

Crystal	RC_vir_XFEL_ CrystFEL		RC_vir_XFEL_ Ideal Crystal		RC_vir_Sync_
Resolution (Å)	55.44–3.5 (3.6–3.5)		55.44–3.5 (3.6–3.5)		50.52–3.6 (3.8–3.6)
*a*, *b*, *c* (Å)	57.9, 84.8, 384.3		57.9, 84.8, 384.3		57.6, 82.9, 382.1
α, β, γ (°)	90, 90, 90		90, 90, 90		90, 90, 90
Space group	*P*2_1_2_1_2_1_		*P*2_1_2_1_2_1_		*P*2_1_2_1_2_1_
Completeness (%)	99.9 (99.8)		99.5 (99.3)		98.9 (92.7)
Unique reflections	24941 (1991)		24932 (1977)		22398 (2986)
Multiplicity	59		59		7
Data processing method	*Process_hkl*	*Partialator*	Correction factor with *b* = 1.05	Correction factor with *b* = 1.19	*XDS*
*I*/σ	3.5 (2.1)	3.9 (2.1)	6.1 (4.8)		4.1 (1.2)
Wilson *B* factor (Å^2^)	26.6	59.6	26.6	59.6	37.6
LLG	3489.50	1565.66	3963.70	4410.41	3333.70
*R*_free_/*R*_work_ (%)	27.4/25.3	31.2/28.6	27.3/25.3	26.6/24.7	28.0/24.2
r.m.s.d. bonds (Å)	0.003	0.004	0.003	0.003	0.016
r.m.s.d. angles (°)	0.712	0.744	0.701	0.725	1.57

**Table 2:** Fitting parameters using a Gaussian and ex-Gaussian distribution for a selected set of reflections from the RC_vir_ data collected at the XFEL *M* = multiplicity for the reflection (*h*, *k*, *l*). χ^2^ = chi-square value calculated by taking twice the difference between the negative of the log-likelihood of a Gaussian and an ex-Gaussian for a single degree of freedom.

*h*	*k*	*l*	Resolution (Å)	*M*	χ^2^	μ_g_	σ_g_	μ_exg_	σ_exg_	τ_exg_
1	21	54	3.50	57	82.6	1022.0	253.6	−532.0	543.3	1594.1
0	22	0	3.85	23	1.1	886.9	281.9	−109.6	1096.5	1036.4
7	17	9	4.25	77	198.5	2355.6	519.0	−763.4	597.2	3172.2
0	0	90	4.25	5	10.7	1491.9	624.9	−228.1	1234.8	2365.5
7	4	5	7.67	119	449.2	7496.7	1330.3	−568.4	621.2	8152.9
0	0	50	7.65	6	0	1.5	211.4	−597.5	511.3	711.5
2	0	5	27.1	158	480.4	7707.6	1044.5	−1070.6	637.1	8871.5
0	0	14	27.3	14	24.1	1331.6	720.7	−1386.7	599.7	2957.4
